# Intermittent Cerebro-Spinal Meningitis

**Published:** 1872-08

**Authors:** George Niemeier


ART. II.—*Intermittent Cerebro-Spinal Meningitis.* *By* GEORGE NIEMEIER, M. D.

On Sunday morning, the 19th of May of the present year, I was called to visit a young married woman, aged about twenty-four years, whom I had safely delivered of a healthy boy on the 14th of March last, and who had been quite well since. I premise that, at the time, small pox was prevalent, though on the decrease; still every week fresh cases of a milder type would break out. Going to bed quite well on Saturday, the 18th, in the middle of the night she suddenly felt very chilly, then hot, and when I saw her she complained of severe frontal headache, pain in the epigastrium, inclination to vomit and actual vomiting, and general lassitude; pulse about one hundred; urine brown as coffee, highly albuminous, temperature decreasing from what it was during the night. Her first question was: "Do you think I will have the small pox?" My answer was: "For all I know you may; we will have to wait and see." I gave her a few Sedlitz powders that day. Seeing her again on the 20th of May, in the morning, she complained of having had a very bad night and high fever. I gave her lemonade. On Tuesday morning, the 21st, when I paid my visit, whom should I see there? Her husband's brother—a young man who is an Eclectic doctor near Toronto, and who, without my knowledge, had been telegraphed for by his brother to see his wife. This young man thought it was bilious remittent fever, and gave her, of course without my consent, Hydrarg. cum Creta, and large doses of opium. I left, but upon urgent solicitation of the husband I returned on the morning of the 26th of May, when I was informed that for four days past, at about six P. M., she was taken with a violent fever and headache, lasting till six o'clock A. M.; and, though weak, she was comparatively well during the day. "What was it?" "My answer was: "Intermittent fever." I gave her four powders composed of Chinioidine, Salicine, Quinine and Sulphate of Beeberine, to be taken at eight, ten, twelve and two o'clock. On Monday morning, the 27th of May, I was informed that the fever the night before had only commenced at about nine o'clock, that she had been delirious and screaming throughout the whole night, and that the fever left her at about five o'clock A. M. She then complained greatly about pains in the neck, indistinct rather, double vision; strabismus, contracted pupils, eyes injected, extreme deafness, and I found the forearms, hands and knees thickly covered with an eruption similar to measles. "What's this?" I was asked. My prompt answer was: "It is Intermittent Fever and Cerebro-Spinal Meningitis." I told them, at the same time, that I was not aware such a thing could be possible; but, nevertheless, it was so. I applied blistering liquid to the temples and behind the ears, five wet cupping glasses and afterwards ice-bags to the nape of the neck, and ice on the head, and the same powders as the day before, with a large dose of Chloral for the night. Thinking it rather singular, I consulted my library when I came home, and found at last in Niemeier's Practice, in the original German, under "Meningitis," a description of an Intermittent Cerebro-Spinal Meningitis, and I was then doubly sure that my diagnosis was correct. On Tuesday morning, the 28th, I was informed that the fever had not returned, and that she slept soundly ten hours after the Chloral. Upper and lower extremities cold, head hot, excessive pain in head and neck, strabismus, quite deaf, tongue moist and soft with white streaks in the center, great prostration, pulse almost regular, eruption more extensive. Ordered hot mustard fomentations around arms and legs, ice-bag as usual, Brom. Pot. and Amon. in large doses four times a day; Chloral for the night in case she does not sleep. For a few days she was progressing as favorably as possible, when, on the 3d day of June, her husband demanded a consultation with another physician, which I refused, telling him that I had not the least doubt or hesitation about the disease or treatment, and if he brought another doctor I would not return. He got another doctor, and I did not return until he came again on the 9th of June, telling me that his wife was dying, and begging me to see her again. I saw her Sunday night, the 9th of June, and then found that extensive Pleuro-pneumonia of the right side had been going on for some days; that she was extremely low, suffering, at the same time, with a bed sore on the right trochanter. Ordered Tinct. of Iodine painted over right chest, hot fomentations, a mixture of Senega and Mur. of Amon, and small doses of Morphine. From that time until now, I have been unremitting in my attendance on her, and what experience and ingenuity could suggest regarding diet and medicines has been done, so that she is now, though weak and emaciated, convalescent.

On the 1st and 2d of June I had three new cases of the same disease, one in town, the two others in the country, all three young men of between eighteen and twenty-one years old, and in each case the intermittent fever commenced, twice not with a quotidian, but with a tertian, type, till, with the third attack, the symptoms of meningitis clearly showed themselves, in each case these young men were even partly able to work during the intermediate days. When I was called, the intermittent type of the disease in two cases had left already, and on account of the extreme rapidity of the pulse I commenced with Tinct. Veratr. Virid. till the pulse was reduced, and then followed up with large doses of Brom. Pot. and Amon., besides cupping and ice-bags. The eruptions, in all of these three cases were large erythematous blotches. They all recovered within ten or twelve days.

I now ask the question, Is cerebro-spinal meningitis an inflammation of the membranes of the brain and spinal cord? I deny it, because the intermittent type, as shown above, excludes the continuous process of inflammation. I may imagine an intermittent congestion, but an intermittent inflammation is a contradiction. Professor Miner, in the March number of the *Buffalo Medical Journal*, page 311, states that he did not find any symptoms of inflammation in the membranes, but that the appearances were normal. I may add that two years ago this last winter I had an epidemic of meningitis, but not of an intermittent character, the first I ever saw, and of twenty-five cases then attended by me none died. They were similarly treated as now.

P. S.—A few days ago I had a case of poisoning in a woman by the seeds of Datura Stramonium, never seen by me before. Symptoms similar to those of Belladonna. Emetic and stimulants, internally and externally, caused recovery in three days.

